# Effects of an integrated safety system for swivel seat arrangements in frontal crash

**DOI:** 10.3389/fbioe.2023.1153265

**Published:** 2023-04-03

**Authors:** Zhao Li, Ruth Gao, Robert McCoy, Hongyu Hu, Lei He, Zhenhai Gao

**Affiliations:** ^1^ State Key Laboratory of Automotive Simulation and Control, Jilin University, Changchun, China; ^2^ School of Public Health, Jilin University, Changchun, China; ^3^ Ford Motor Company, Dearborn, MI, United States

**Keywords:** autonomous vehicle, integrated safety system, swivel seat arrangement, occupant kinematics, injury risk

## Abstract

**Objective:** Autonomous vehicles (Avs) have paved the way for the arrangement of swivel seats in vehicles, which could pose a challenge to traditional safety systems. The integration of automated emergency braking (AEB) and pre-pretension (PPT) seatbelts improves protection for a vehicle’s occupant. The objective of this study is to explore the control strategies of an integrated safety system for swiveled seating orientations.

**Methods:** Occupant restraints were examined in various seating configurations using a single-seat model with a seat-mounted seatbelt. Seat orientation was set at different angles, from −45° to 45° with 15° increments. A pretension was used on the shoulder belt to represent an active belt force cooperating with AEB. A generic full frontal vehicle pulse of 20 mph was applied to the sled. The occupant’s kinematics response under various integrated safety system control strategies was analyzed by extracting a head pre-crash kinematics envelope. The injury values were calculated for various seating directions with or without an integrated safety system at the collision speed of 20 mph.

**Results:** In a lateral movement, the excursions of the dummy head were 100 mm and 70 mm in the global coordinate system for negative and positive seat orientations, respectively. In the axial movement, the head traveled 150 mm and 180 mm in the global coordinate system for positive and negative seating directions, respectively. The 3-point seatbelt did not restrain the occupant symmetrically. The occupant experienced greater *y*-axis excursion and smaller *x*-axis excursion in the negative seat position. Various integrated safety system control strategies led to significant differences in head movement in the *y* direction. The integrated safety system reduced the occupant’s potential injury risks in different seating positions. When the AEB and PPT were activated, the absolute HIC15, brain injury criteria (BrIC), neck injury (Nij), and chest deflection were reduced in most seating directions. However, the pre-crash increased the injury risks at some seating positions.

**Conclusion:** The pre-pretension seatbelt could reduce the occupant’s forward movement in the rotating seat positions in a pre-crash period. The occupant’s pre-crash motion envelope was generated, which could be beneficial to future restraint systems and vehicle interior design. The integrated safety system could reduce injuries in different seating orientations.

## 1 Introduction

Autonomous vehicle technology has undergone rapid development in the last decade. However, several issues must be solved before the large-scale usage of AVs. Based on statistical data, 90% of crashes are fully or partially caused by human error (Aufrère et al., 2003). AVs have the great potential to eliminate human error and thus reduce vehicle crashes. AVs are anticipated to not be involved in crashes, however, AVs may still crash, especially during a transition period (Sivak and Schoettle, 2015).

AVs provide a great opportunity and freedom to redesign the occupant compartment, interior system, and restraint system to provide and protect occupants in various seating configurations (SAE, 2016). AVs are characterized by higher design freedom of vehicle interiors, namely, flexible seat positioning, orientations, and reclining (Jorlöv et al., 2017; SAE, 2019). Vehicle manufacturers could consider developing such solutions, while still having to ensure the protection of all occupants in the alternative seating arrangements. Current restraint systems are designed and optimized on standards and strict protocols and will not be effective in crash scenarios based on flexible seating arrangements (Adam and Untaroi, 2011; Yamada et al., 2016; Bosma et al., 2017; Luttenberger et al., 2018).

These new seating configurations are prompting a new area of research in passenger safety (Subit et al., 2017; McMurry et al., 2018; Hasija et al., 2019; Bohman et al., 2020). A seat not facing the front faces significant differences from the point of view of conventional safety research. Most research investigated the effects of seat orientation on occupant kinematics focusing on the interaction between the occupant and the seat belt system, whereas interactions with airbag systems and internal structures were excluded (Kitagawa et al., 2017; Gayzik et al., 2018). The results showed that the combination of seat belts and seat orientation was critical for controlling occupant kinematics. The protective effect of a restraint system might vary under different seating configurations.

It was reported that the forward collision warning system can identify impending impacts and warn the driver to take action about 1.5 s before the collision (Kusano and Gabler, 2012). Kitagawa et al. (2017) and Wu et al. (2020) investigated the concept of an active seat rotation strategy that changes seat orientation during a pre-collision timeframe. A baffle structure must be added to guide the dummy rotated with the seat, such a modification requires significant follow-up work for verification. A seat-mounted airbag provides protection regardless of how an occupant is seated (Matsushita et al., 2019). A life cell airbag resembles a protective cocoon once it is fully activated (Autoliv, 2018). These new devices provide ideas for occupant protection of AVs and completely change the design of restraint systems.

An impact could be detected much earlier by advanced sensors. The ability to strategically reposition the vehicle and occupant due to advanced sensors was not considered in the reviewed studies. A pre-crash emergency braking would provoke occupant movement, which could lead to an interaction with the passive safety system. Acceptance corridor was developed based on head trajectories resulting from pre-crash maneuvres (Battaglia et al., 2013). Diederich et al. (2021) extracted the occupants’ head kinematics envelope during braking for rotated seat arrangement wearing a lap-belted and 3-point seat belt. The integration of AEB and PPT seatbelts in frontal seat orientation was evaluated. The result showed that the integration of PPT and AEB reduced injury risks further than AEB alone and that a higher pretention force would reduce the rib fracture risk while increasing concussion risk (Osth et al., 2015; Savino et al., 2016).

A pre-crash maneuver will cause the occupant to be out of position and thus reduce the protection of the restraint system in case of a crash. The current focus on safety is shifted toward integrated safety systems to further enhance occupant protection. Capturing the occupant’s kinematics in a swiveled seat arrangement during the pre-crash phase is critical to define the vehicle interiors as well as the requirements of future restrain systems. This study investigated the effect of the integrated safety system on the trajectory and injury of an occupant for swiveled seating orientations. Future restraint systems and new vehicle interior layouts can be designed according to the occupant’s position in an AV during the pre-crash phase. The objectives of this study are to:

a) Investigate occupant kinematics in rotated seat arrangements during the AEB stage
b) Investigate occupant kinematics in various seat belt arrangements during the AEB stage
c) Investigate the injury risk influenced by the integrated safety system

## 2 Materials and methods

The interaction between an occupant and an integrated safety system was simulated using a simplified swivel seating compartment and a human body finite element (FE) model (THUMS, Version 4.0, AM50 occupant model). The effects of AEB and PPT were evaluated by simulating a pre-crash braking scenario for PPT seatbelt configuration.

### 2.1 Vehicle collision simulation model

**FIGURE 1:**
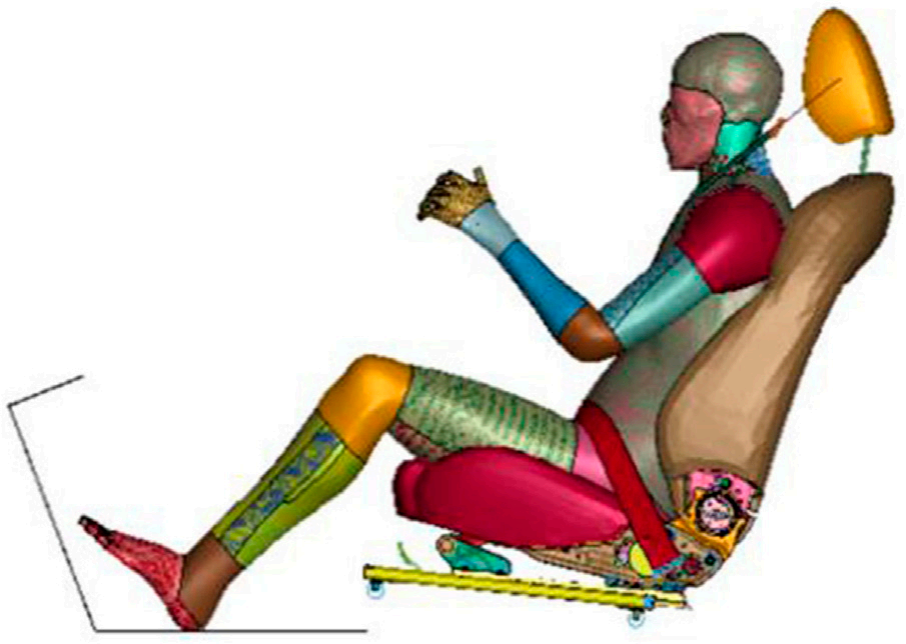
Frontal collision sled model setup.

**FIGURE 2:**
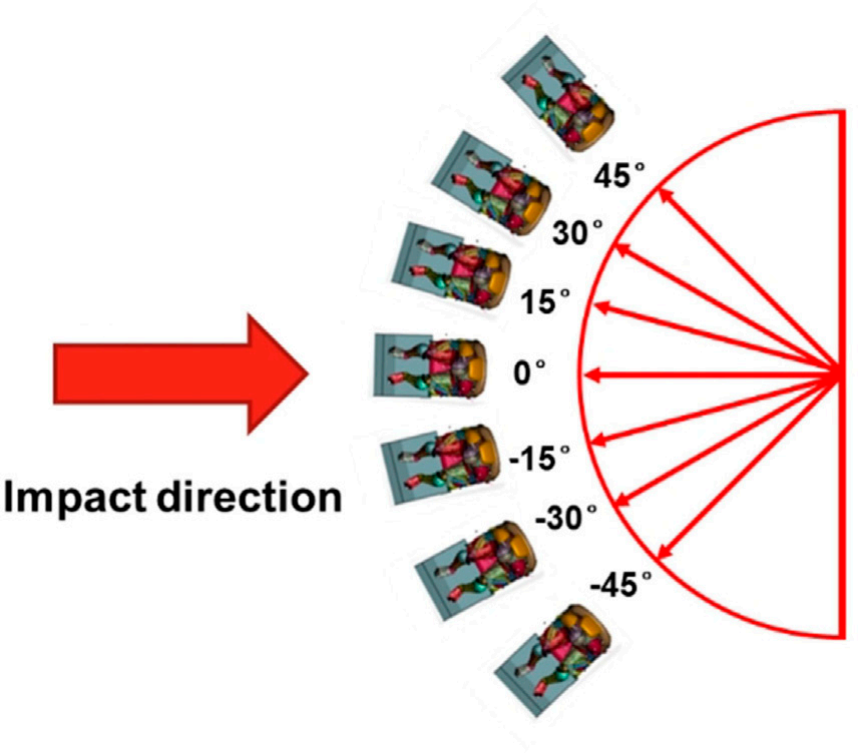
Seat rotation angle definition.

The steering wheel and airbags were not included, and the occupant was only restrained by a 3-point seat belt. A deformable seat model from a production vehicle was used. The seat back angle was 22.5°. The seat track was connected to the floor through four bolts, as shown in Figure 1. The sled provides support for both feet and the angle of the sled is 110°. Seat-integrated restraints with a standard three-point belt can be adapted in AVs with swiveled seat configurations. The D-ring of the seat belt was fixed to the seatback instead of a B-pillar, and the position of the D-ring was determined according to the traditional vehicle. AVs allow a 360-degree arrangement of seats with different crash mechanisms, such as 90° for a side impact and 180° for a rear impact. This study focused on frontal collision, and the seat orientations were set from −45° to 45° with 15° increments, as shown in Figure 2 (Kitagawa et al., 2017; Wu et al., 2020).

### 2.2 Validation of PPT

**FIGURE 3:**
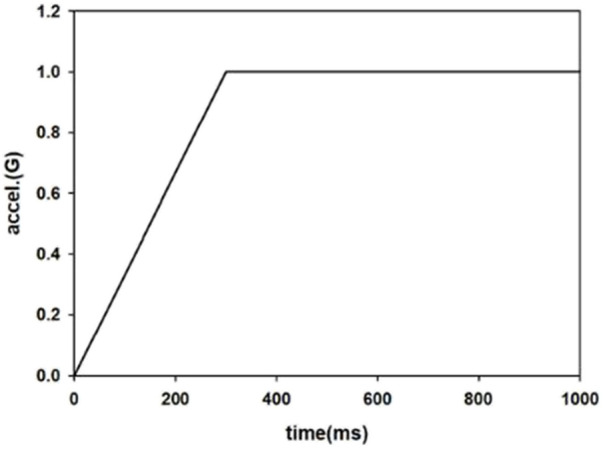
Pulse of AEB.

**FIGURE 4:**
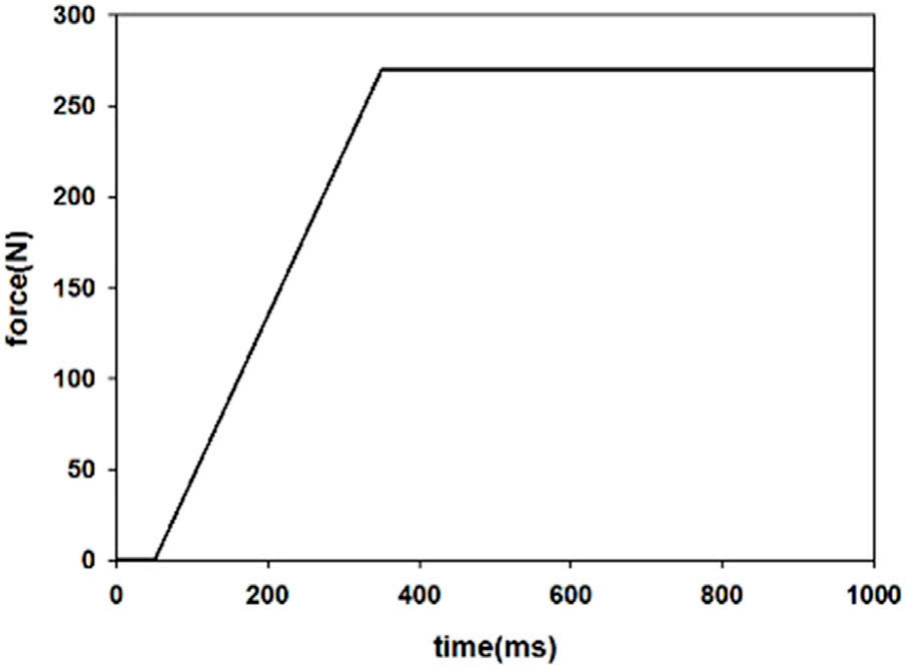
Force curve of pre-pretensioner.

The braking pulse was raised to the maximum of 1G within 300 ms and then kept constant until the end of the AEB phase at 1000 ms (Figure 3). A pretensioner was used on the shoulder belt to represent a pre-pretensioner that cooperated with AEB. The pull-in is given as a function of time to prevent the occupant from moving away from the initial position, and a 50 ms delay was defined before the pre-pretensioner activated, as shown in Figure 4. When the belt forces generated exceeded the pretensioner force limit (270 N), the retractor would play a role to hold the occupant. The active seat belt obtained from this study was compared with a volunteer test from the literature with similar parameters of active seat belt and pre-crash braking (Wen, 2019).

### 2.3 Integrated safety system and crash

Both pre-crash and crash phases were simulated in the same run, without restarting the simulation. The total time for the simulations was 1,200 ms, which was composed of a 1000 ms pre-crash duration and a 200 ms crash. The occupant’s kinematics response was analyzed as a function of seat rotation under the integration of AEB and PPT by extracting the head center of gravity (CG)’s pre-crash kinematics envelope.

**FIGURE 5:**
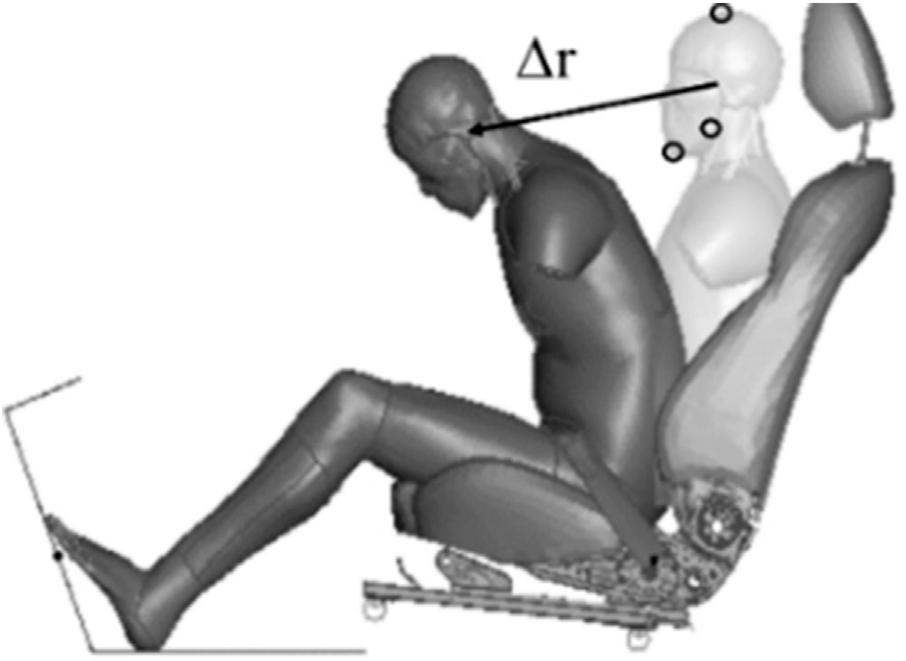
Relative displacement of the occupant head.

The coordinates of the initial dummy head CG were given in a Cartesian coordinate system and were considered as the origin. The head CG deviated from the origin of coordinates in the *x*-axis, *y*-axis, and *z*-axis was defined as the head movement (Figure 5). The head CG in the xy-plane was evaluated to form the occupant kinematics’ envelope and define the enclosed movement space in that plane. The top of the head, chin, left cheek, and right cheek can be chosen to represent the envelope of head movement in a real vehicle interior design.

The occupant’s kinematics was studied with various seat orientations in the AEB stage. A head trajectory envelope in the AEB stage was extracted considering an active 3-point seat belt to define the occupants’ head motion space.

To investigate the effect of different integrated safety parameters on an occupant, the active seat belt pre-pretensioner delay was changed from 50 ms to 0 ms and 100 ms (Kent et al., 2007; Battaglia et al., 2013). The occupant movement response was compared for three configurations.

**FIGURE 6:**
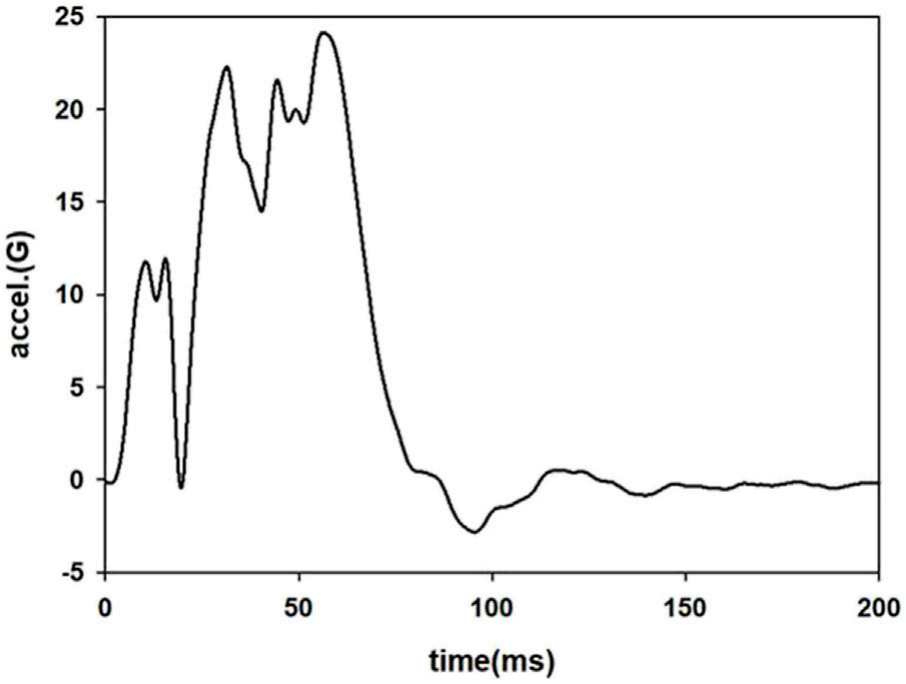
Crash pulse.

The vehicle slowed down from 38 mph to approximately 20 mph during pre-crash. A 20 mph generic frontal vehicle pulse was applied to the sled model, as shown in Figure 6. The injury values were calculated for various seating directions with an integrated safety system.

Another simulation of a 20 mph crash was conducted without pre-crash. The force limits of the seat belt retractor and pretensioner were 4 kN and 90 N, respectively.

To assess the risk of injury in a crash simulation, injury criteria and risk curve recommended by NHTSA research were used in this work. The nodal data at the head CG was output to calculate brain injury (HIC and BrIC). A cross-section was created at the intervertebral disc between C1 and C2 to measure the axial load and bending moment with respect to a local coordinate system, which was used to calculate neck injury (Nij). The distance change between the sternum and T8 was measured to present chest deformation.

## 3 Results

### 3.1 Validation of PPT seatbelts

**FIGURE 7:**
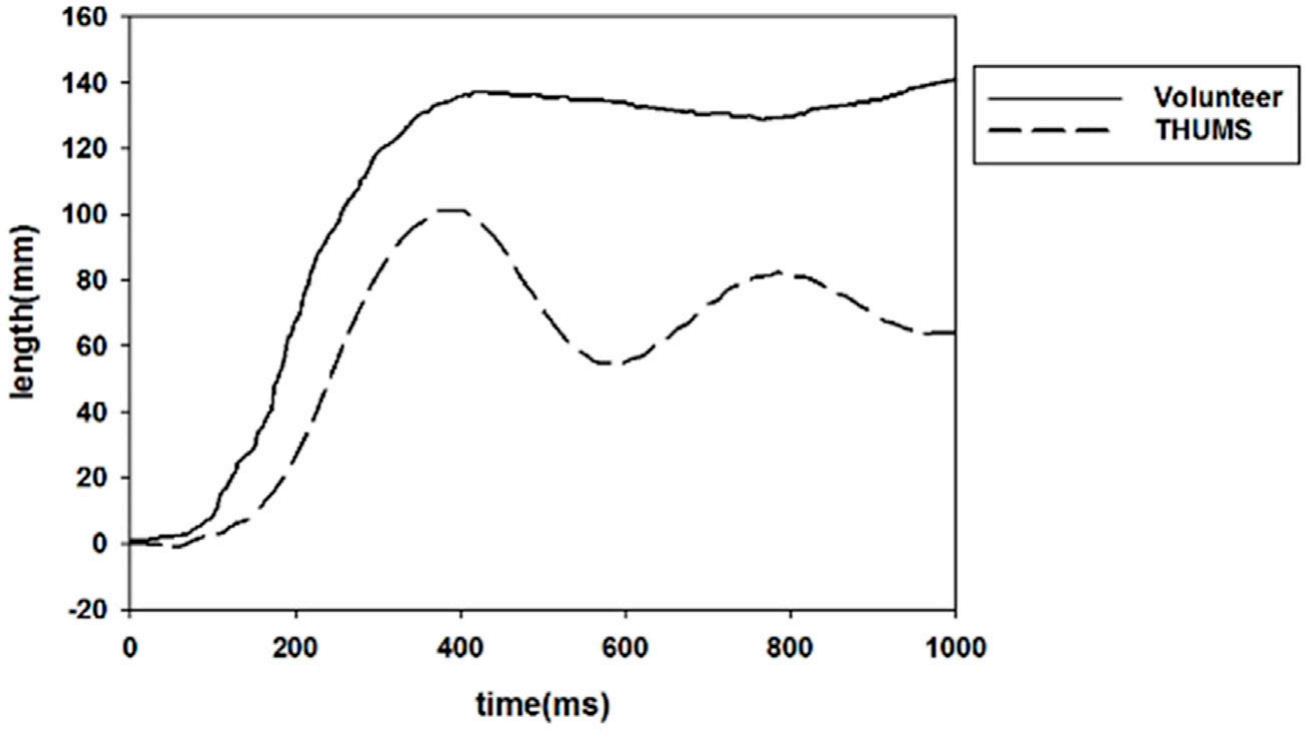
Neck movement during pre-crash.

**FIGURE 8:**
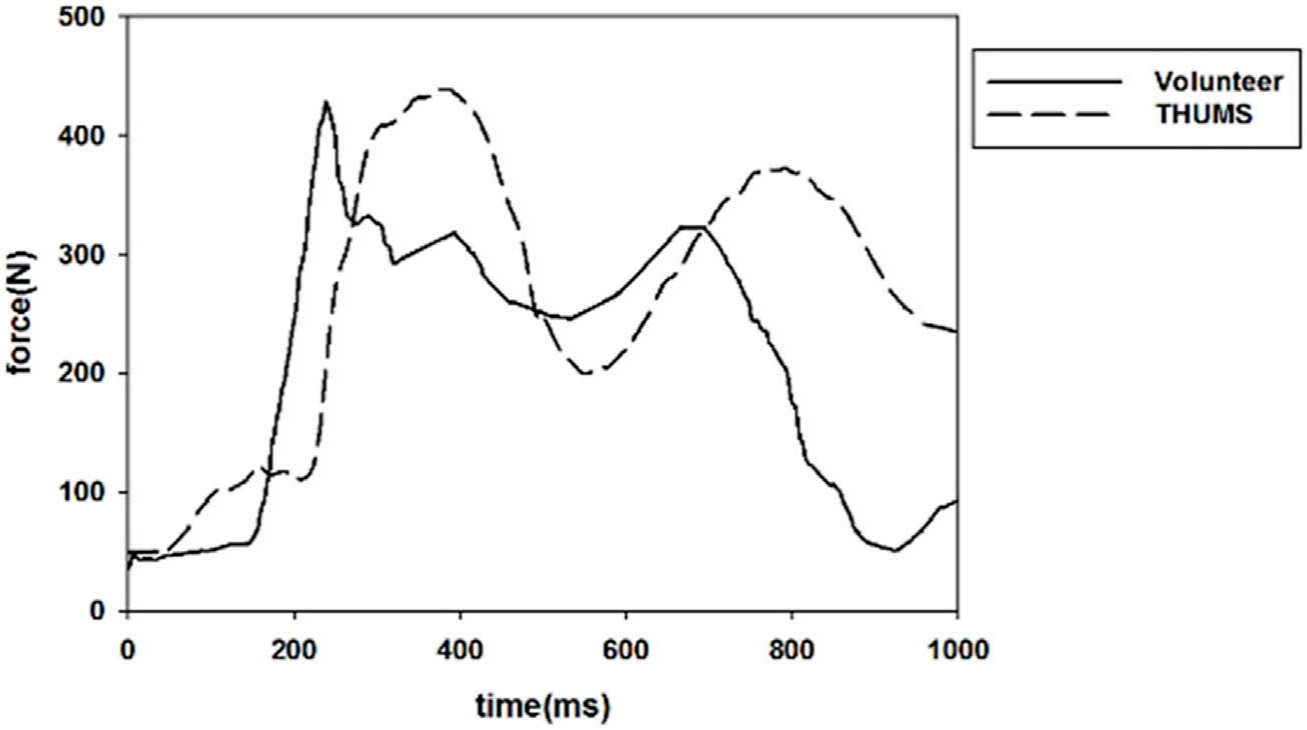
Shoulder belt force during pre-crash.

By simulating a pre-crash, the dynamic response of the occupant in a pre-crash was obtained and compared with the neck displacement and shoulder belt force in a volunteer test, as shown in Figures 7, 8. The result shows that the THUMS model maintains the same trend as the volunteer in neck motion and shoulder belt force curve.

The THUMS model exhibited a large fluctuation in the neck displacement, which decreased rapidly and then maintained a small fluctuation. The shoulder belt force also produced a corresponding fluctuation. In the initial phase of braking, the seat belt force was in high agreement with the volunteer response values in terms of both trend and value. In the simulation, the shoulder belt force of the active seat belt configuration still provided a large force to restrain the occupant in the late braking period.

### 3.2 Occupant’s kinematics in the AEB phase

**FIGURE 9:**
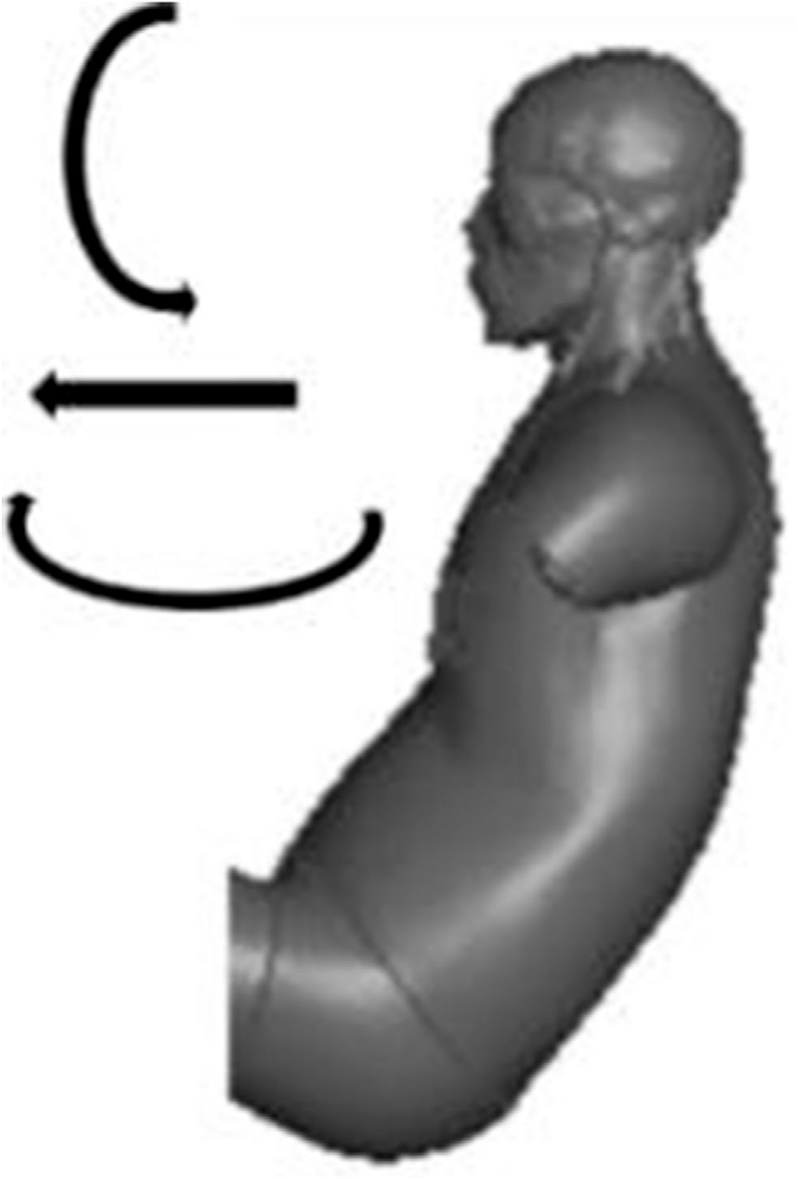
Occupant motion in pre-crash.

In the case of swiveled seating arrangement, during an emergency pre-crash maneuver, the dominant motion of the occupant is a combination of translation along the vehicle’s *x*-axis and rotation about the vehicle’s *y*-axis and *z*-axis, as illustrated in Figure 9.

**FIGURE 10:**
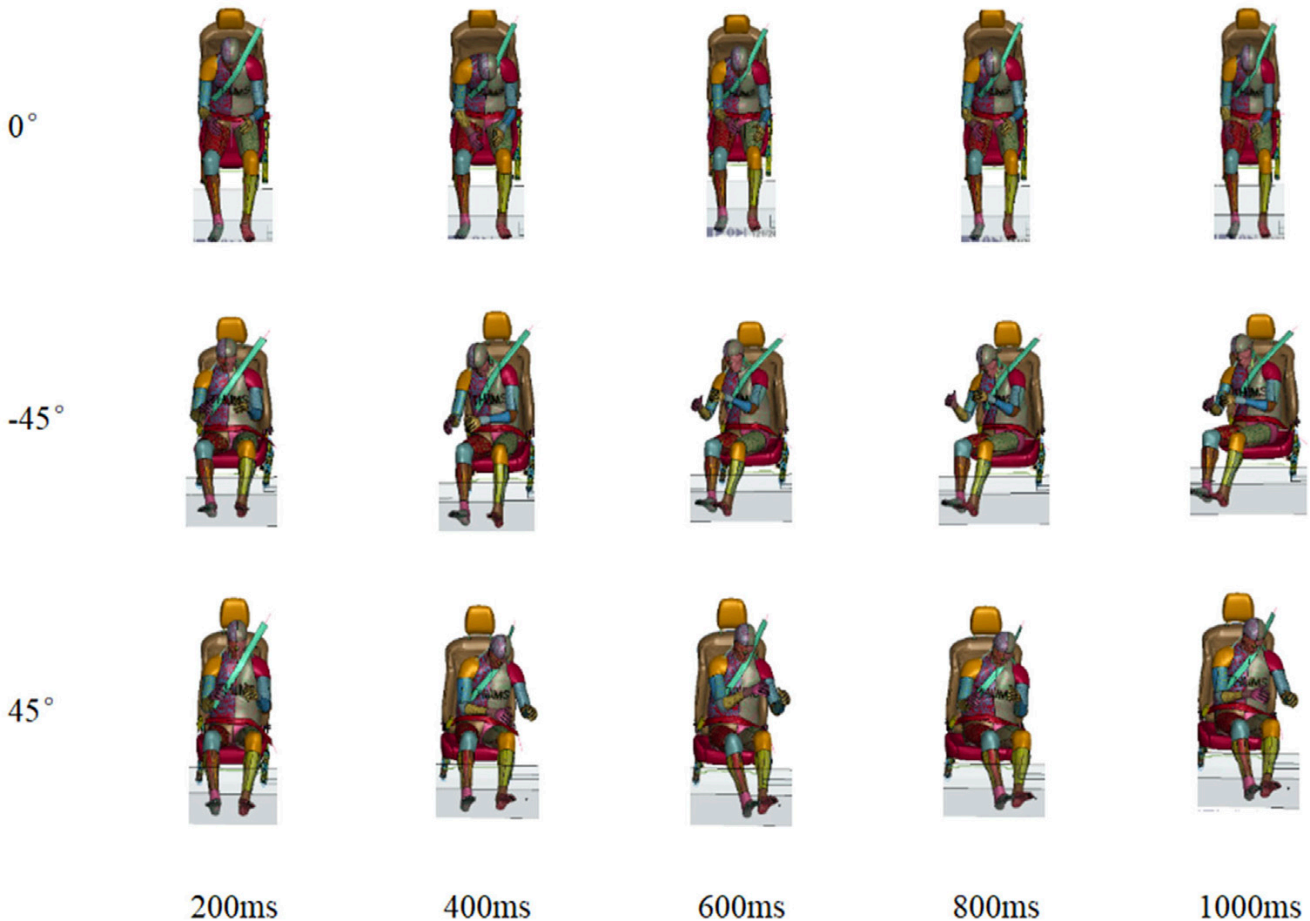
Occupant kinematics in 0°, −45° and 45° seat orientations.

The occupant kinematics during AEB was quite different among various seating orientations, which can be divided into two phases. In phase 1, the occupant moved forward due to the deceleration to the maximum displacement of approximately 400 ms. In phase 2 (t > 400 ms), the pre-tensioned seatbelt pulled the occupant’s upper body back, as shown in Figure 10.

**FIGURE 11:**
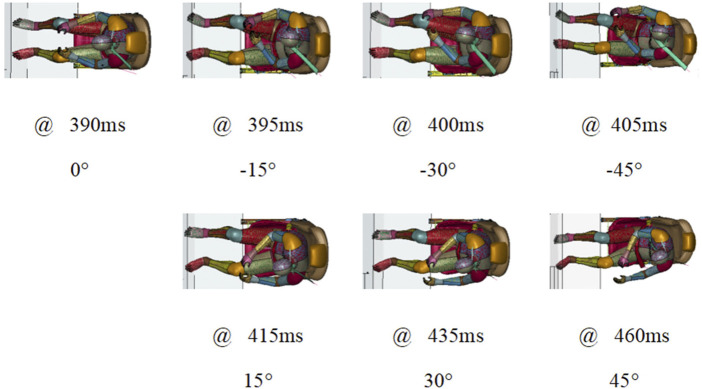
Top view of occupant’s maximum displacement at various seating arrangement.

The occupant motion for various seating orientations in the AEB stage could be reduced by an active seatbelt. The occupant kinematics at maximum displacement can be observed in Figure 11. The maximum displacement was reached at approximately 400 ms. The moments of maximum head displacement for the left seat orientations were earlier than those for the right seat orientations.

**FIGURE 12:**
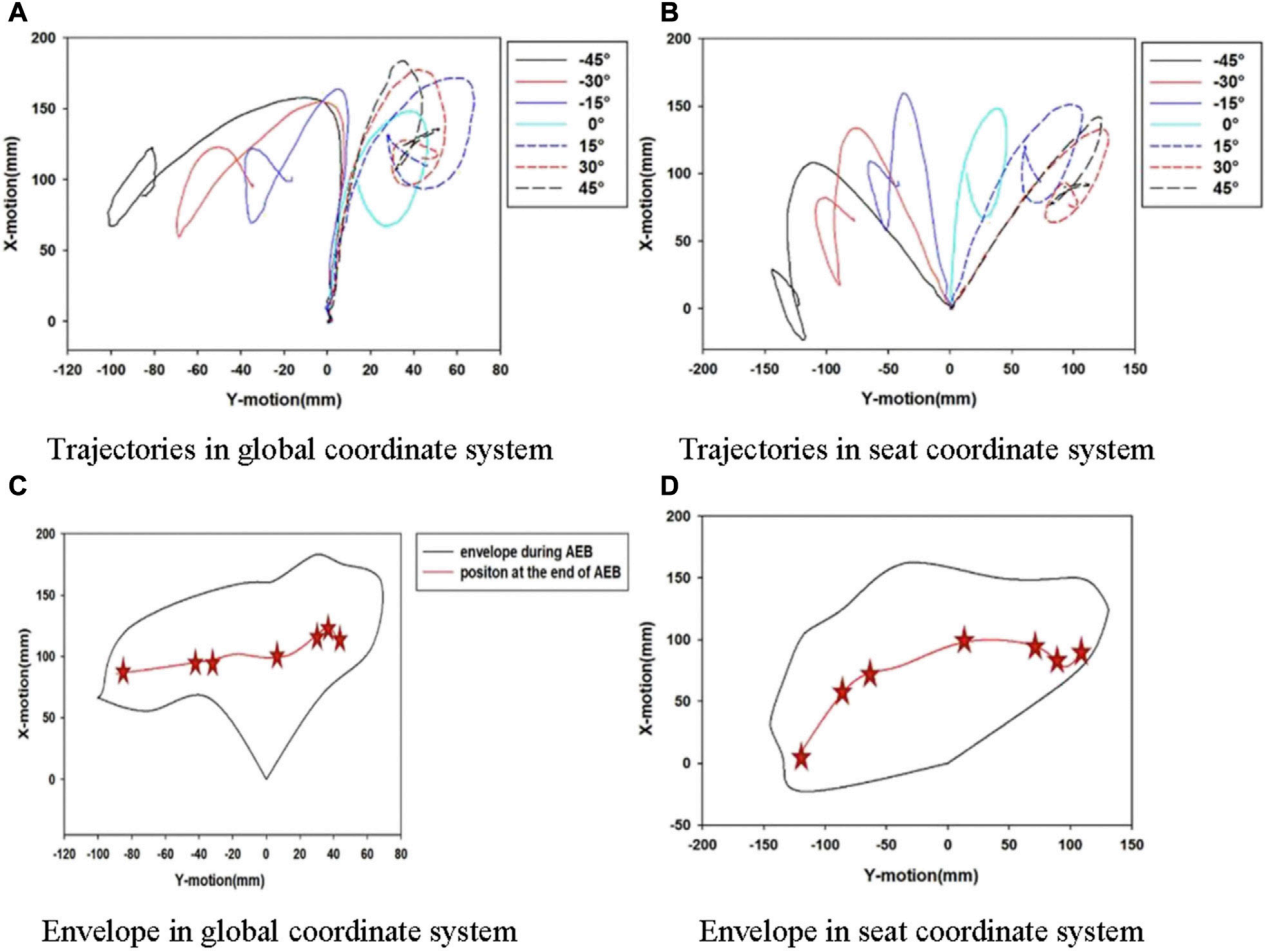
Head CG trajectories and envelope for all seating arrangement. **(A)** Trajectories in global coordinate system **(B)** Trajectories in seat coordinate system **(C)** Envelope in global coordinate system **(D)** Envelope in seat coordinate system.

The head movement trajectory can be found in Figures 12A, B for all seating positions. In a lateral movement, the excursions of the head were 100 mm and 70 mm in the global coordinate system for negative and positive seat orientations, and the values were 150 mm and 130 mm in the seat coordinate system, respectively. In an axial movement, the head traveled 150 mm and 180 mm in the global coordinate system for positive and negative seating directions, and 160 mm and 150 mm in the seat coordinate system, respectively. At different seat angles, the movement of the head in the *x* direction was not significantly different compared to that in the *y* direction. The head movement was significantly different when the seat turned to the left than when the seat turned to the right. The largest left movement happened at the −45° seating position, while the largest right movement was found at the 30° seating position. The 3-point seat belt seems to have a significant influence on the occupants’ motion when the occupants are in the right seating orientations.

A head trajectory envelope in the xy-plane with the rotated seat is shown in Figures 12C, D. The occupant experienced a great *y*-axis excursion and a small *x*-axis excursion in the left seat position. The head position at the end of pre-crash is critical to the airbag design, for instance, the shape and deployment time. The kinematics envelope can provide some guidance to avoid interference between an occupant and vehicle interior components.

To investigate the effect of different integrated safety parameters on an occupant, an active seat belt pre-pretensioner delay was changed from 50 ms to 0 ms and 100 ms. The occupant movement response was compared for three configurations.

**FIGURE 13:**
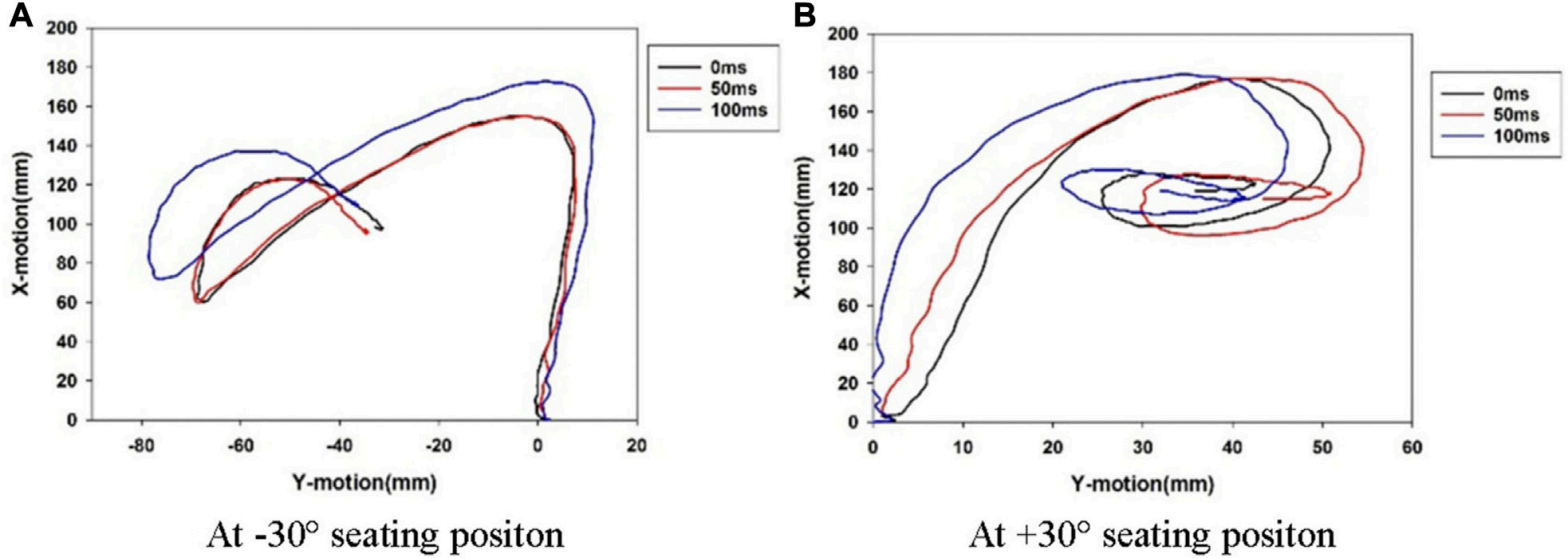
Head CG trajectories for different active seat belt delay time at ±30° seating arrangement. **(A)** At −30° seating positon **(B)** At +30° seating positon.

Figure 13 illustrates the head CG movement trajectory for 0 ms, 50 ms, and 100 ms active pretension seatbelt at ±30° seating arrangement. The delay time affected the head displacement, while the trend of the head trajectory had little effect.

**FIGURE 14:**
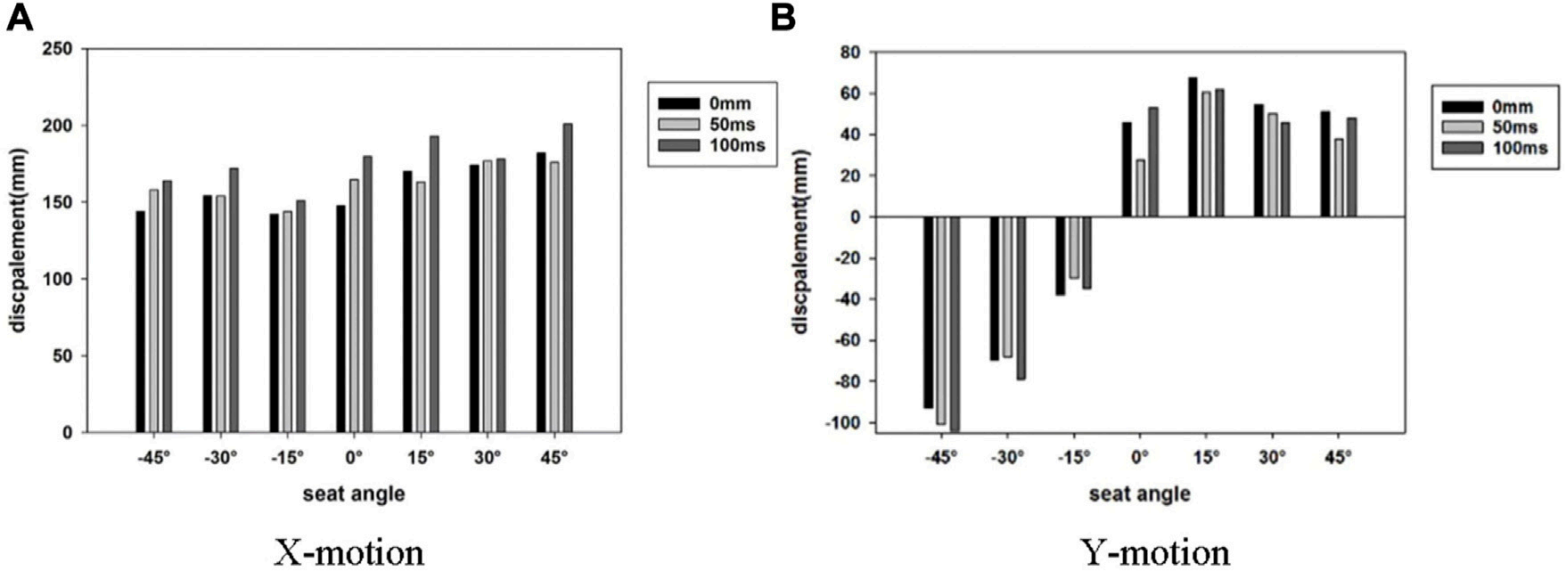
Head maximum displacement in different active seatbelt delay time. **(A)** X-motion **(B)** Y-motion.

The head maximum resultant xy-displacement is shown in Figure 14 in different active seatbelt delay times for all seat rotation angles. In the *x* direction, the difference between the head displacement of the 0 ms and 50 ms delays was not significant, while the head displacement of the 100 ms delay time was significantly larger than the other two delay times. The head movement in the *y* direction was complex. The 100 ms delay caused the smallest head displacement at the ±15° seat orientation, while the 0 ms delay generated the largest head displacement at the +15°, +30°, and +45° seat positions. The delay of 50 ms performed well at all angles, especially at 0°.

### 3.3 Occupant injury risk in crash

**FIGURE 15:**
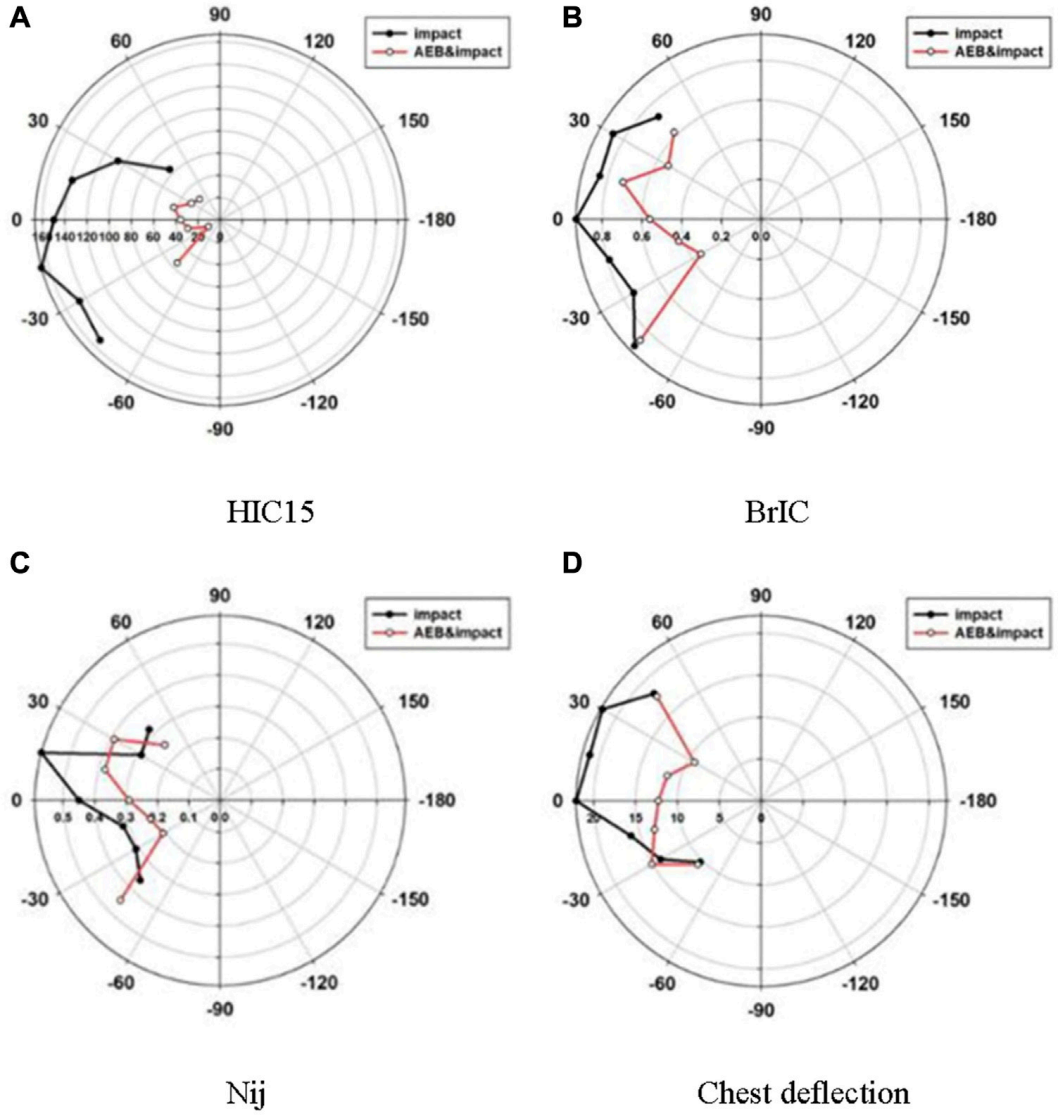
Comparison of injury number in impacts with and without AEB. **(A)** HIC15 **(B)** BrIC **(C)** Nij **(D)** Chest deflection.

Compared to the impact without pre-crash, the integrated safety system helped to reduce the potential injury in different seating positions in most cases. The comparison of injury numbers for the impacts with and without pre-crash is shown in Figure 15.

The HIC15 ranged from 64 to 167 in an impact without pre-crash, while the values were distributed between 12 and 55 in the case of an integrated safety system. The integrated safety system reduced HIC15 significantly, even though all the values were far below the head injury threshold of 700.

An integrated safety system reduced the brain injury criteria (BrIC) in all seating orientations. The BrIC ranged from 0.73 to 0.9 in the impacts without pre-crash, while BrIC ranged from 0.35 to 0.86 in the case of AEB. The integrated safety system reduced BrIC significantly, except for the case at −45°. In the case of a pre-crash, the highest BrIC value was obtained at the −45° and −30° seating positions, respectively. The same seat orientations led to significant differences in BrIC in impacts with pre-crash and without pre-crash. 

The effect of an integrated safety system on Nij is not clear. A pre-crash reduced the Nij in most cases, except at −45° and +30° seating positions. The Nij values in an impact without AEB ranged from 0.29 to 0.59, and the Nij values in the case of AEB ranged from 0.21 to 0.45. Nij could be reduced in cases of impact when using an integrated safety system in AEB compared to the impact without AEB. However, the pre-crash increased Nij at the −45° and 30° seat orientations by 15% and 12%, respectively.

An integrated safety system could reduce chest deflection in most seating orientations. There was no obvious discrepancy in chest deflection observed for various seating orientations using AEB before an impact. The chest deflections ranged from 10.3 mm to 20.1 mm in an impact without AEB, while ranging from 9.2 mm to 17.6 mm in the case of AEB. The integrated safety system reduced chest deflections significantly when the seat orientated from −15° to +30°. The highest chest deflection happened at the 0° seat position and the lowest value was found at the −45° seat position in a no AEB impact. In the case of AEB, the corresponding seating orientations were +45° and +30°, respectively. The similar seat orientations of +45° and +30° led to a great difference in chest compression. The chest deflections were reduced for the impact with AEB compared with the impact without AEB for the 0° and ±15° seating orientations.

## 4 Discussion

This study performed FE simulations to evaluate the effect of AEB and PPT seatbelts on occupant kinematics during pre-crash and the injury risks in frontal car crashes in swiveled seating positions.

To eliminate the effects of other factors, the occupants were restrained by a seatbelt, which differs from the conventional seating configuration with the restraint of an airbag and a windshield. Without restraint ahead, the occupant has longer travel space in a floor, seat, and seatbelt configuration.

The displacement of the occupant’s head (∼150 mm) was larger compared to a volunteer test (∼100 mm) at the 0° seating position (Olafsdottir et al., 2013). Investigation of volunteer response to braking events has generally been done in connection with the relaxed and braced muscle states at various acceleration levels. In a volunteer pre-crash study, the peak forward head excursion caused by the acceleration pulse showed to be very subject-specific, the values were in the range of 34–514 mm (Erlinger et al., 2022). Considering active muscle forces could be complicated, the occupant model used in this study was a passive human body model. The THUMS model did not have a high agreement with the volunteer’s response values in both movement trend and displacement values, however, the differences were still acceptable.

In the constraint and interior design concept of AVs, the occupant movement affected by the pre-crash maneuver should be considered. By lowering the vehicle speed, AEB helps to avoid an impact or reduce the occupant injury risk in an unavoidable impact. While the PPT seatbelt tenses the shoulder belt in a pre-crash phase, it can reduce the forward excursion significantly and hold back the occupant to his/her initial position.

Occupant motion in a pre-crash phase can alter the initial occupant posture in a crash event and affect the kinematics and loads of the occupant during the follow-up crash event. Injury risk can be changed with the occupant out of position posture due to a pre-crash (Bose et al., 2010). Therefore, it is important to accurately predict the occupant posture in pre-crash with an integrated safety system.

A PPT seatbelt was found to be effective by improving the coupling of an occupant to a seat structure. An angular dependence of occupant restraint was found in the study, as shown in Figures 11, 12. The shoulder belt did not wrap symmetrically around the occupant. The shoulder belt engaged the shoulder firmly when the seat rotated to the right. The shoulder belt slipped off from the shoulder when the seat rotated to the left. However, in both left and right directions the lap belt helped the occupant to remain in the seat. The head experienced a longer lateral motion when the seat was in the left seating position, as shown in Figure 12.

The head displacement with respect to the seating direction showed similar profiles for the PPT delay times, as shown in Figure 13. This suggests that though a 3-point belt is effective in restraining the occupant in a rotated seat in a pre-crash scenario, there is still potential to improve its performance by tuning the time of releasing preload. In addition, the PPT pulse is an important factor, which will be investigated in future research.

Based on the passenger’s position at the end of the pre-crash in Figure 14, the optimal control strategy can be applied to different angles of the seat. For seats at −45° and −30°, the seatbelt delay of 0 ms can minimize the dislocation of passengers. In the case of 15° and 45°, the delay of 50 ms was the best. When the seat was at 0° and −15°, the control strategy depended on whether the *x*-direction or *y*-direction displacement was given priority. At 30° of seat orientation, the 100 ms seat belt delay produced the smallest *y*-direction displacement, while the three-time delays did not lead to much difference in the *x*-direction displacement.

In a crash without an integrated safety system, there is a delay and slack of the seatbelt (Pipkorn and Wass, 2017) and the occupant travels forward when the seatbelt works. An integrated safety system reduces occupant injury risk in most situations. AEB slows down the vehicle and a PPT triggers to prevent passengers from staying in their original position. The seatbelt works ahead of the crash and enhances the performance of protection. It should be noted that, due to the absence of a steering wheel and an airbag in the simulation, the significantly roomier space allowed the occupants to have sufficient damping, while a standard crash with a steering wheel and an airbag limits the damping In the absence of an airbag, the head rotated around T1, generating a higher angular velocity when the body was restrained and obtaining the high BrIC value. The reduction in BrIC was not significant as that of HIC15. In some seat positions, the integrated safety system enhanced the occupant’s risk of neck injury. The shoulder slipped off from the seatbelt causing upper body flexion in the left seating position. Chest compression was mainly caused by a seat belt. At a small degree of seat orientation, a seat belt covered well the center of the chest during a full frontal impact. However, at a large degree of seat orientation, the seat belt moved up toward the neck and away from the chest during a full frontal impact. The integrated safety system allowed the seat belt to act earlier on the chest, extending the belt’s working time and reducing the force on the belt. In the case of a pre-crash, the values of HIC15, BrIC, Nij, and chest deflection were quite different at the −45° and −30° seating positions. Similar seat orientations led to significant differences in injury risk. The reason for this result needs to be further studied.

The animation of the collision phase shows that the baffle influences the movement of the lower limbs. When the seat was at 0°, the lower limbs moved forward with the body, and the lower limbs retracted toward the trunk after the feet hit the baffle. When the seat was rotated, the trunk and lower limb movements appeared to be separate. The torso was relatively coherent with the seat under the seat belt restraint, while the unrestrained lower limbs still moved forward. The lower limbs were thrown around the hip joint and retracted after the feet touched the baffle.

## 5 Conclusion

Frontal impact simulations with 1G emergency braking deceleration and active seat belt configurations were performed to study the effect of an integrated safety system in a swiveled seating arrangement. The kinematics and injury risks following impact were also evaluated. The occupant’s head pre-crash motion envelope was generated, which can be beneficial for future restraint systems and vehicle interior design. The occupant being out of position is also important for any airbag systems design, such as an airbag mounted on the seat, deploying over the head, or from the vehicle roof.

1) The occupant in a right-rotated seat endured more shoulder engagement and exhibited a smaller head excursion.
2) The occupant’s excursion during a pre-crash could be improved by coupling a PPT seatbelt release time.
3) It was observed that a pre-crash could affect the following impact. In an unavoidable impact, an integrated safety system could reduce the injuries of an occupant in various seating orientations.

## Data Availability

The raw data supporting the conclusion of this article will be made available by the authors, without undue reservation.
